# Two-Level 3D Column-like Nanofilms with Hexagonally–Packed Tantalum Fabricated via Anodizing of Al/Nb and Al/Ta Layers—A Potential Nano-Optical Biosensor

**DOI:** 10.3390/ma16030993

**Published:** 2023-01-21

**Authors:** Andrei Pligovka, Andrei Lazavenka, Ulyana Turavets, Alexander Hoha, Marco Salerno

**Affiliations:** 1 Research and Development Laboratory 4.10 “Nanotechnologies”, Belarusian State University of Informatics and Radioelectronics, 6 P. Brovki Str., 220013 Minsk, Belarus; 2 Department of Micro- and Nanoelectronics, Belarusian State University of Informatics and Radioelectronics, 6 P. Brovki Str., 220013 Minsk, Belarus; 3 Institute for Globally Distributed Open Research and Education (IGDORE), Institute for Materials Science, Max Bergmann Center of Biomaterials, Technische Universität Dresden, 27 Budapester Str., 010169 Dresden, Germany

**Keywords:** anodization, TaO_2_, Ta_2_O_3_, NbO_2_, aluminum oxide, niobium oxide, tantalum oxide, valve metal, biomedicine, COVID-19

## Abstract

Reanodizing metal underlayers through porous anodic alumina has already been used extensively to fabricate ordered columns of different metal oxides. Here, we present similar 3D multilayered nanostructures with unprecedented complexity. Two-level 3D column-like nanofilms have been synthesized by anodizing an Al/Nb metal layer in aqueous oxalic acid for forming the first level, and an Al/Ta layer in aqueous tartaric acid for forming the second level of the structure. Both levels were then reanodized in aqueous boric acid. The Ta layer deposited on partially dissolved porous anodic alumina of the first level, with protruding tops of niobia columns, acquired a unique hexagonally-packed structure. The morphology of the first and second levels was determined using scanning electron microscopy. Prolonged etching for 24 h in a 50%wt aqueous phosphoric acid was used to remove the porous anodic alumina. The formation mechanism of aluminum phosphates on the second-level columns in the process of long-time cold etching is considered. The model for the growth of columns on a Ta hexagonally-packed structure of the second level is proposed and described. The described approach can be applied to create 3D two- or three-level column-like systems from various valve metals (Ta, Nb, W, Hf, V, Ti), their combinations and alloys, with adjustable column sizes and scaling. The results of optical simulation show a high sensitivity of two-level column-like 3D nanofilms to biomedical objects and liquids. Among potential applications of these two-level column-like 3D nanofilms are photonic crystals for full-color displays, chemical sensors and biosensor, solar cells and thermoresponsive shape memory polymers.

## 1. Introduction

One-level column-like nanofilms of anodic tantala and niobia formed by anodizing of Al/Ta and Al/Nb bilayer systems have been studied for many years [1,2,3,4]. Great interest in such unique nanostructured films is caused by their widespread use in capacitors [5,6], sensors [7,8], semiconductors, thermistors and varistor structures [9,10], hydrophobic and oleophobic coatings [11] and, recently, in optical and photonic devices [12,13]. Column-like nanofilms find practical application alone as well as in combination with porous anodic alumina (PAA). Depending on the required parameters, a type of structure is selected to provide the necessary set of electro-physical or spectral-optical properties of the thin-film system.

The formation process of one-level column-like nanofilms involves electrochemical anodizing of sputter-deposited Al/Nb and Al/Ta layers, which proceeds with the PAA formation and subsequent anodizing of the metal layer through the pores of the PAA [9,14]. As a result of high-voltage reanodizing, the tantala and niobia from underlying metal fills the PAA pores [15,16]. The resulting nanofilms are composed of metal oxide nanocolumns protruding from a thin continuous layer of valve metal and embedded in PAA.

Numerous studies over the last decades [1,2,3,5,6,8,9,11,14,17,18,19,20,21,22,23,24,25,26,27,28,29,30,31,32] have shown that the microgeometry, composition and properties of column-like nanofilms are directly affected by anodizing conditions, such as anodizing voltage, anodic current density, pH, temperature, and electrolyte composition (Table 1).

**Figure 1:**
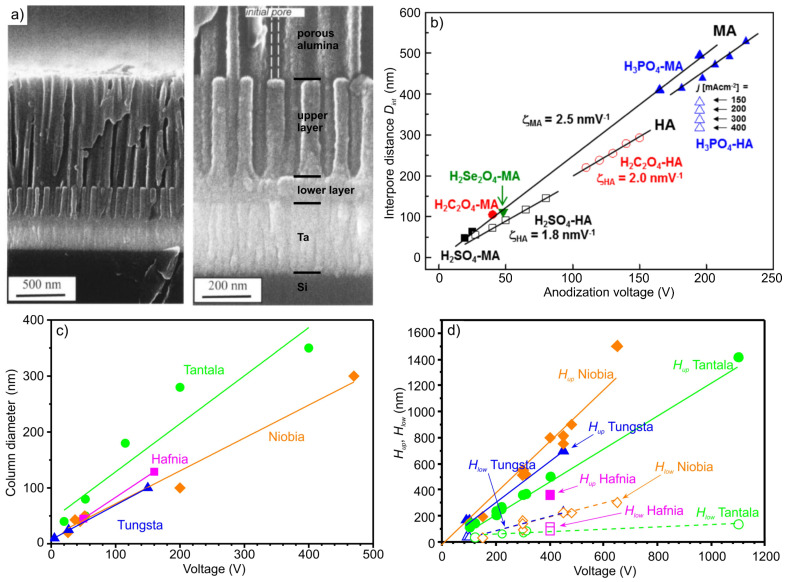
(**a**) SEM images of one-level column-like nanofilm morphology reproduced from Ref. [27]. (**b**) Anodizing voltage influence on PAA cell size (interpore distance) by using H_2_SO_4_ (black symbols), H_2_C_2_O_4_ (red symbols), H_2_SeO_4_ (green symbol), and H_3_PO_4_ (blue symbols), MA: mild anodizing, filled symbols, and HA: hard anodizing, void symbols; reproduced from Ref. [29]. (**c**,**d**) Size dependence of tantala, niobia, tungsta and hafnia continuous layer, column diameter and height from reanodizing voltage taken from Refs. [1,2,3,5,6,8,9,11,14,17,18,19,20,21,22,23,24,25,26,27,28,29,30,31,32].

Based on generally accepted architecture [27], one-level column-like nanofilms are distinguished, which have the following characteristics: column diameters *D* and height *H_up_*, as well as continuous valve metal oxide thickness *H_low_* (Figure 1a). Column diameters *D* of first-level column-like 3D nanofilms depend on the pore diameters of PAA (Figure 1b), and therefore on aluminum anodizing voltage. The column diameters always exceed the pore sizes and linearly depend on the anodizing voltage (Figure 1a–c), and the column height *H_up_* and continuous valve metal oxide thickness *H_low_* is proportional to the voltage of reanodizing (Figure 1d) [1,2,3,5,6,8,9,11,14,17,18,19,20,21,22,23,24,25,26,27,28,29,30,31,32]. Figure 1b–d show that it is possible to form intercolumn distance of 13–1100 nm (Figure 1b), column diameters of 10–350 nm (Figure 1c), continuous valve metal oxide heights of 35–440 nm and column heights up to 1500 nm (Figure 1d), which overlap the range of optical wavelength across the UV–VIS–NIR spectrum. Values of magnetic and dielectric constant for tantala, niobia, tungsta and hafnia are displayed in Table 1 [33,34,35,36,37]. Therefore, Figure 1b,c show a wide possibility to form one-, two- and multi-level column-like nanofilms with various periodicity, height, and magnetic and dielectric constant. Additionally, to improve the regularity of the PAA pores, the use of two-stage [38] or three-stage anodizing methods [14] and of stamps [39] will allow the formation of highly ordered defect-free structures. The combination of nanostructured composite layers with regular and controlled microgeometry, and predictable electrophysical and spectral-optical properties, opens up prospects for obtaining fundamentally new functional materials with unusual and yet unexplored characteristics.

**Table 1:** Magnetic and dielectric constant of tantala, niobia, hafnia, tungsta.

Oxide	*µ_r_*	*ε_r_*
**NbO_2_**	1.0000003 [34]	10 [40]
**Nb_2_O_5_**	0.9999998 [33]	32–45 [35,36]
**TaO_2_**	1.0000032 [34]	27.5 [41]
**Ta_2_O_5_**	0.9999994 [33]	Ta_2_O_5_ (α)$\varepsilon_{11}=\varepsilon_{22}=30$, $\varepsilon_{33}=65$ [33] Ta_2_O_5_ (β) *ε_r_* = 24 [33]
**WO_2_**	1.0000029 [33]	5–12 [42]
**WO_3_**	0.9999995 [33]	300 [33]
**HfO_2_**	0.9999989 [33]	16–19 [37]

In this work, the fundamental possibility of obtaining two-level column-like nanofilms with 3D architectures using the method of anodizing a valve metal layer through PAA was shown in Refs. [1,13,14]. In the first layer, a niobium underlayer and PAA with small pore sizes were used. In the second layer, a tantalum underlayer and PAA with large pore sizes were used. The two-level column-like 3D nanofilms were examined by scanning electron microscopy (SEM) and energy-dispersive X-ray spectroscopy (EDX), providing the deepest information about their morphology and microstructure. Simulations of the optical responses of the two-level 3D column-like nanofilms to the presence of biomedical objects and liquids are also shown.

## 2. Materials and Methods

### 2.1. First-Level Column-like Anodic Niobia Nanofilm Preparation

**Figure 2:**
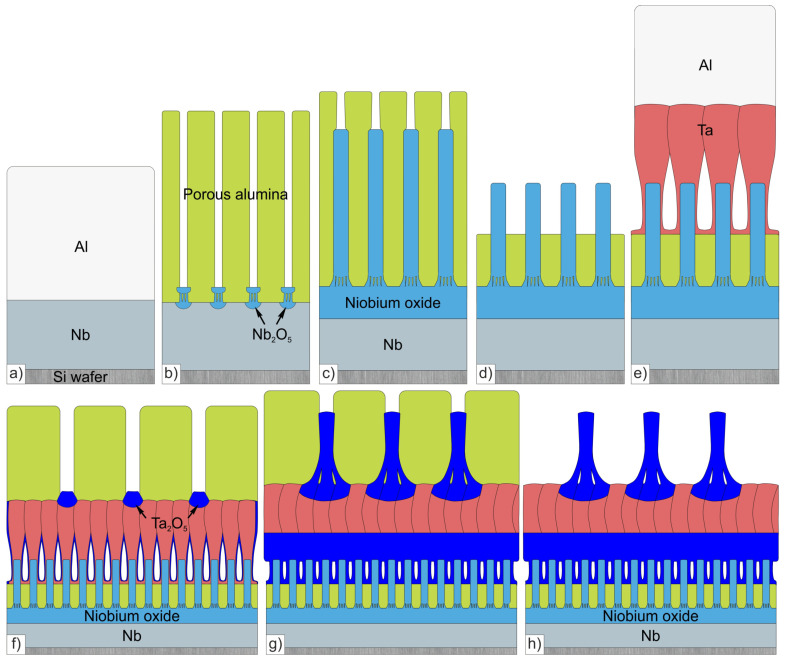
Schematic diagram showing the main steps for forming two-level column-like with hexagonally-packed tantalum 3D nanofilms via anodizing Al/Nb and Al/Ta metal bilayers on Si wafer: (**a**) sputter-deposition of Al/Nb bilayer, (**b**) anodizing the Al layer to form PAA with small pore sizes, (**c**) reanodizing of the Nb-layer through PAA with small pore sizes, (**d**) chemical removal of part of the niobia nanocolumns and PAA, (**e**) sputter-deposition of Al/Ta bilayer, (**f**) anodizing the Al layer to form PAA with large pore sizes, (**g**) reanodizing of the Ta-underlayer through PAA with large pore sizes, (**h**) selective dissolution of topmost PAA.

A 100 mm diameter polished wafer of *n*-type, (111) single crystal silicon was the starting substrate. Then, 300 nm thick niobium was sputter-coated on the former. Then, 1200-nm-thick Al was coated onto the Nb as shown in Figure 2a. The metals were deposited using DC magnetron sputtering. The silicon wafer provides a microscopically flat substrate surface and is convenient for film fracture preparation studies for SEM investigations of cross-fracturing.

The whole wafers with the Al/Nb layers were anodized in a specially designed cylindrical two-electrode cell made of polytetrafluoroethylene (PTFE). The Al/Nb/Si wafer was placed horizontally in the cell and a PTFE ring was fastened tightly to the aluminum surface, and the area available for anodizing was ~63 cm^2^. In the initial experiments, the anodizing potential was measured with a calomel electrode, by probe and agar bridge, such that we could determine the potential drop between electrodes and cathodic voltage. This value was ∼0.4 V, i.e., negligible compared to the used anodizing voltage. A controllable lab supply Keysight N5752A (Keysight Technologies Inc., Santa Rosa, CA, USA) was used as the anodizing unit, which is capable of sweeping the voltage up to 600 V, controlled by a personal computer (PC) with homemade software written in LabVIEW. Time dependent process variables, such as anodizing voltage and current, were registered by a programmable digital multimeter 34,470 A (Keysight Technologies Inc., Santa Rosa, CA, USA) used to record the voltage-time responses, controlled by a PC with R&D Lab 4.10 developed software. The bulk electrolyte temperature was 23 °C, maintained typically within ±1 °C of the set value.

The chemicals for preparation of the nanofilms were supplied by Belaquilion (Minsk, Belarus) additional liability company and Sigma-Aldrich, Inc. (Darmstadt, Germany). The process of the first-level column-like anodic niobia 3D nanofilm forming is outlined in Figure 2a–d. The first anodizing step is carried out in 0.4 M aqueous oxalic acid at 53 V until growth of a PAA-film, resulting in consumption of the residual aluminum down to the niobium surface (Figure 2b). The given anodizing conditions resulted in a PAA thickness of ∼1740 nm, taking into account a Pilling–Bedworth ratio (PBR) of ~1.45 [14]. After the alumina barrier touched the niobium, local oxidation occurred across the alumina pores and continued until nanosized niobia embryo-like columns appeared at the interface; see Figure 2b (for details check Refs. [16,30]).

The second anodizing step is reanodizing of the formed system in 0.5 M H_3_BO_3_ and 0.05 M Na_2_B_4_O_7_ aqueous solution (pH 7.4) at 23 °C by sweeping the voltage at a constant rate of 0.1 V s^−1^ from zero to the maximum achievable anodic value. As reported before [30], high-voltage reanodizing of the initially anodized Al/Nb bilayer sample consumes the remaining niobium metal locally under the pores, with the formation of niobia nanocolumns penetrating into the small PAA pores, as shown in Figure 2c. The extent to which the pores are filled by growing niobia depends strikingly on the anodic value outlined in Figure 1d. Obviously, under proper conditions, the oxide columns fill in the pores along their way, arriving at the film top at the end of reanodizing. Considering Figure 1d, which describes the relationship between column height and reanodizing voltage, the maximum achievable anodic value should be ∼800 V. However, under these conditions, in particular when using boric solution, such a voltage could not be achieved. Reanodizing was finished at a voltage of ∼450 V. Current fluctuations and random micro-sparks over the specimen surface were observed around this voltage. The sparks and current fluctuations may be associated with the phenomenon of electrical breakdown during the formation of the barrier film on the niobium underlayer, terminating normal oxide growth. The height of the niobia columns at the anodic value of 450 V was 770–800 nm [9,10]. The first-level surface was chemically prepared for deposition of a bilayer Al/Ta system. Initially, the surface was treated in a 30% aqueous HF solution at 23 °C for 1 min, then in peroxide-ammonia aqueous solution NH_4_OH:H_2_O_2_:H_2_O in proportion 1:1:5 at 75–95 °C for 20 min. As a result of this treatment, the height of the pillars and the porous oxide decreased by almost one half. It should be noted that, initially, according to the experimental plan, such a processing was not supposed to be carried out, and before it began the first level was planarized in the hot H_3_PO_4_ and H_2_CrO_4_ mixture (hereafter the selective etchant) at 60 °C for 28 min.

### 2.2. Second-Level Column-like Anodic Tantala Nanofilm Preparation

The process of forming the second level of column-like 3D nanofilm of anodic tantala is presented in Figure 2e–h. A 500 nm thick layer of tantalum, followed by a 2000 nm thick layer of aluminum layer, were sputter-deposited onto the wafer by radio-frequency magnetron method (Figure 2e). The second anodizing step was carried out at constant current density of 6 mA·cm^−2^ in 0.2 M aqueous tartaric acid. During this stage, the second-level aluminum layer was fully converted into PAA, with pores larger than the former PAA layer (Figure 2f). An array of bulge-like tantala nanosized protrusions was formed in the niobium layer, penetrating the alumina barrier layer under the pores. Then, the tantalum underlayer was reanodized locally through the PAA at constant current density of 400–600 µA·cm^−2^, corresponding to a voltage of up to 450 V, in a borate buffer solution 0.5 M H_3_BO_3_/0.05 M Na_2_B_4_O_7_ (pH 7.4) at 23 °C, so that tantala continued to grow along the pores (Figure 2g). The extent of pore filling by growing tantala, as previously shown, depends strikingly upon the formation voltage. After formation of niobia and tantala nanocolumns, the PAA was selectively dissolved in 50% aqueous H_3_PO_4_ at 50 °C for 10 min (Figure 2h). Then, for complete removal of the PAA, a long etching in the same solution at 23 °C for 24 h was carried out.

### 2.3. Nanofilms Analysis

The nanofilms in a SEM S-4800 (Hitachi High-Technologies Corp., Tokyo, Japan) operated at 10–15 kV were observed. Nanofilms were coated with a thermally evaporated 3 nm thick gold film for reducing the electrical charging effects.

The nanofilm composition was determined by energy EDX on a Quantex 200 (Bruker Nano GmbH, Berlin, Germany) with a resolution of 125 eV.

### 2.4. Data Processing and Optical Simulations

Curve-fittings and graphical characteristics were developed using OriginPro 2019 (OriginLab Corp., Northampton, MA, USA), and calculations are performed using mathematical tables Excel 2016 (Microsoft Corp., Mountain View, CA, USA).

The FDTD simulations were performed with available software FDTD Solutions ver. 8.24.2502 (Lumerical Inc., Vancouver, BC, Canada) on a PC. The dielectric material properties, permittivity:

(1) $$\sqrt{\varepsilon }=n+i\cdot k$$

where n+i⋅k is the complex refractive index (RI), were taken from Ref. [43] for the 250–2500 nm wavelength region as the default setting. A mode source was used for the simulation. Boundary conditions were set as perfectly matched layers in *X*, *Y*, *Z* directions. Mesh type settings were set as custom non-uniform where maximum mesh step was set as 10 nm for *dx*, *dy*, *dz*.

## 3. Results and Discussion

### 3.1. First-Level Anodic Niobia Nanofilm Morphology

**Figure 3:**
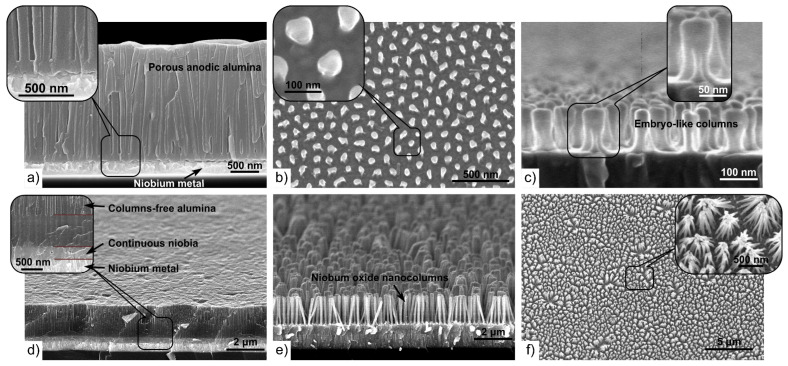
SEM cross-fractured (**a**,**c**–**e**) and top surface (**d**,**f**) views of the first-level column-like anodic niobia 3D nanofilms grown on the Al/Nb bilayer in 0.4 M aqueous H_2_C_2_O_4_ via (**a**–**c**) anodizing at 53 V followed by (**d**–**f**) reanodizing at 450 V. Images shown in (**b**,**c**,**e**,**f**) were taken after the PAA layer had been dissolved.

Figure 3a SEM shows surface and cross-section images of the anodized Al/Nb bilayer.

The PAA pores can be clearly seen in the image. The average diameter of the observed pore outlets is ∼20 nm, with an average interpore distance D_int_ of ∼130 nm, as shown in Figure 1b. One can see in Figure 3b,c the top and tilted views of the embryo-like niobia nanocolumns after the dissolution of PAA in the selective etchant. At the initial stage, the embryo-like columns consist of thin roots emerging from the niobium continuous layer and connecting into a single trunk. These structures, formed at low voltages, have skittle-like shape [16] (Figure 3b,c), but, with increasing anodizing voltage, are transformed into nanocolumns [13].

Comparing the embryo-like nanocolumns with the PAA pores, one can see that their width (∼50 nm) is bigger than the pores diameter (∼20 nm). The embryo-like columns have a uniform height of ∼110 nm from the niobium surface, which exceeds the thickness of the alumina barrier layer (∼55 nm). Moreover, the lower part of the embryo-like columns consists of 4–6 roots and its length (∼60 nm) is comparable to the barrier layer thickness. Based on this, it can be concluded that the roots are nanochannels formed in the barrier layer and grow into the single stem in the lower part of the pores. Table 2 presents the morphological parameter values of the first-level column-like 3D nanofilms fabricated by anodizing the Al/Nb (Figure 2a–d).

The inner surface of the pore walls is the loose part of the PAA doped with electrolyte ions, containing bound water and cationic inclusions, and differs in microstructure and composition from the anodic oxide in the cell walls middle and in the barrier layer [44]. Niobium ions, moving under the electric field action through nanochannels in the barrier layer, penetrate the pore cavity [28] and, oxidizing, compress the “loose” non-stoichiometric part of the PAA, expanding the pores and forming niobia nanocolumns increased in diameter [16,30]. A representative SEM image of the cross-fracture of the Al/Nb sample after sequential anodizing and reanodizing is shown in Figure 3d. The nanocolumns penetrating the alumina barrier and coming up in the pores have relatively enlarged tops and appear to be located on a continuous oxide base. Figure 3e,f show SEM views of the cross- fracturing and surfaces of the sample after dissolving the PAA layer in the selective etchant, with different resolution. The first-level column-like 3D nanofilms are the nanocolumn array standing on a continuous niobia film. The columns exhibit diameters of ~40 nm, exceeding the size of the initial PAA pores, and uniform height of ~444 nm.

### 3.2. Morphology and Structure of Sputter-Deposited Al/Ta Bilayer

**Figure 4:**
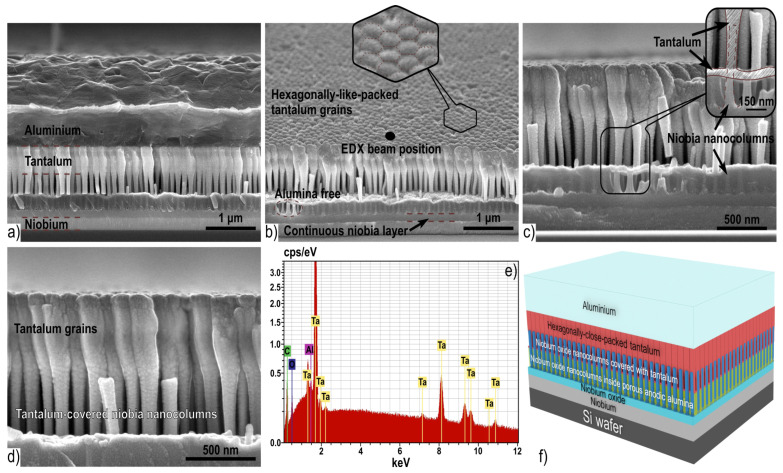
SEM cross-fractured (**a**–**d**) and 3D schematic views (**f**) of the Al (2000 nm)/hexagonally-packed Ta (500 nm) bilayer system, sputter-deposited on the first-level column-like anodic niobia 3D nanofilms. (**e**) EDX point analysis of the hexagonally-packed tantalum.

Figure 4a displays a top surface SEM image of the Al/Nb/Si-wafer after anodizing and reanodizing, partial dissolution of the PAA and niobia, and sputter-deposition of the Al/Ta bilayer, as described in Figure 2e.

During the deposition process, tantalum covered the wall tops of the niobia columns, and penetrated through the space between the columns and formed a thin layer on the alumina surface. In Figure 4c, the inset shows these places where the tantalum coating formed during its deposition. Over the time, during growth, the tantalum film closed at the tops of the niobia columns, further thickening up to forming a continuous film. As a result, the whole composition structure, which is shown in the micrograph of Figure 4a, consists of the following layers: Si wafer—niobium metal—continuous niobia—columns of anodic niobia penetrating the PAA—columns of anodic niobia penetrating the thin tantalum film—columns of anodic niobia covered with a thin tantalum film—tantalum—aluminum.

In Figure 4b–d, it is clearly shown that the tantalum film, deposited on the tops of the niobia columns, has pronounced nanostructure. Moreover, the lower part of the tantalum film is the continuation of the columns (Figure 4c), which—gradually expanding and connecting with each other—form the upper continuous and grainy part of the film (Figure 4d). The tantalum grains are hexagonally-packed and form a smooth surface with slightly pronounced relief (Figure 4b).

### 3.3. Second-Level Anodic Tantala Nanofilm Morphology

**Figure 5:**
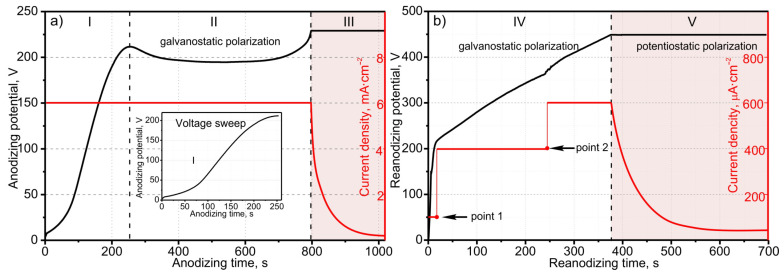
Current- and voltage-time responses grown from the second-level column-like 3D nanofilm during the PAA anodizing (**a**) in 0.2 M aqueous C_4_H_6_O_6_ followed by reanodizing (**b**) in 0.5 M aqueous H_3_BO_3_ and 0.05 M aqueous borax of an Al (2000 nm)/hexagonally-packed Ta (500 nm) bilayer sputter-deposited on the first-level column-like anodic niobia. The marked stages are: I—nucleation and beginning of a steady-state growth of pores, II—steady-state pore growth (at 200 V), III—complete alumina, nucleation, and development of tantala embryos (Figure 2f), IV—galvanostatic reanodizing the Ta underlayer up to 450 V (Figure 2g), V—galvanodynamic-potentiostatic polarization at 450 V (current decay). The profiles have been presented in two separate panels, for the sake of clarity.

Anodizing of the sputter-deposited nanostructured two-layer Al/Ta system, in which the tantalum film consists of hexagonally-packed grains, was carried out under standard conditions, at a current density of 6 mA·cm^−2^. As usual, when anodizing such a thin-film system, the process went through three stages. Figure 5a shows the potential–time and current–density–time responses recorded during the galvanostatic–potentiostatic anodizing of the Al/Ta (hexagonally-packed) bilayer in the 0.2 M aqueous C_4_H_6_O_6_. During the galvanostatic polarization (stages I and II), PAA grows with constant rate at the steady-state potential of ~196 V, reaching the nanostructured tantalum layer. When the barrier layer touches the nanostructured tantalum and the potential begins to rise, the power supply is switched into the constant-potential regime, preventing the oxide from further growth (stage III), which is accompanied by the current decay.

The potential–time and current–time responses for reanodizing up to 450 V are shown in Figure 5b. When the anodic polarization is turned on at low current, a voltage surge occurs around 200 V to the value of the potential stabilization during initial anodizing (stage IV). Then, the current (point 1) increases to ensure the stable growth of tantala. When the voltage growth rate begins to decline, the current (Point 2) still increases twofold, to keep the growth rate of anodic potential uniform. Thus, the voltage increase during reanodizing at a constant current indicates field-stimulated growth of tantala; after voltage stabilization (stage V), the smooth decrease in current is the result of ion transport completion across the oxide film and the columns growth in the PAA pores.

**Figure 6:**
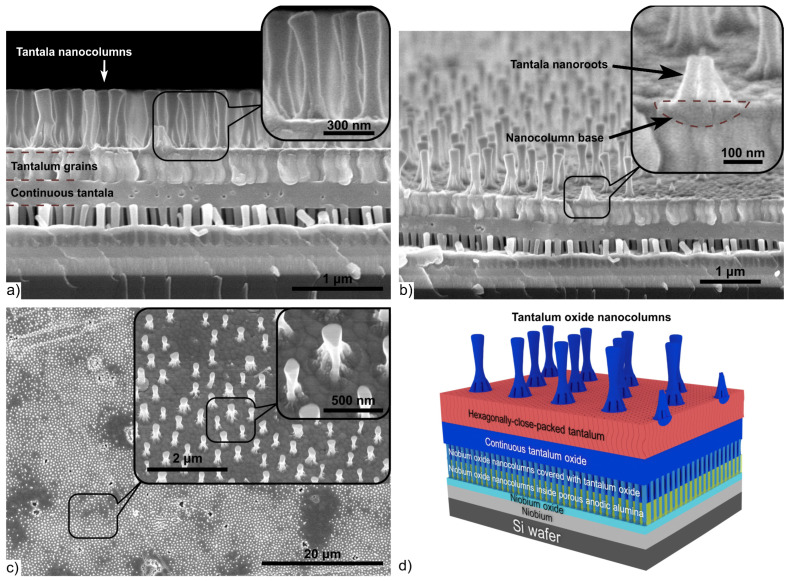
SEM cross-fractured (**a**,**b**), surface (**c**) and 3D schematic views (**d**) of the second-level column-like niobia 3D nanofilm with hexagonally-packed tantala. Images shown in (**a**–**c**) were taken after the second-level PAA layer had been dissolved away.

Figure 6 shows an SEM image and sketch of the Al/Nb/Al/Ta/Si-wafer after anodizing and reanodizing of the second level and chemical dissolution of PAA, as described in Figure 2h.

The nanofilm shown in the micrograph in Figure 6a consists of the following layers, bottom-up: Si wafer—niobium—continuous niobia—columns of niobia penetrating the PAA pores—columns of niobia penetrating a layer of continuous tantala—columns of niobia covered with a thin film of continuous tantala—top of columns of anodic niobia penetrating a layer of continuous tantala—nanostructured tantalum—columns of tantala without PAA, which had been dissolved (“alumina-free” surface). The morphological parameters of second-level tantala nanofilm fabricated by anodizing of the Al/Ta on the anodized Al/Nb structure are shown in Table 3.

**Figure 7:**
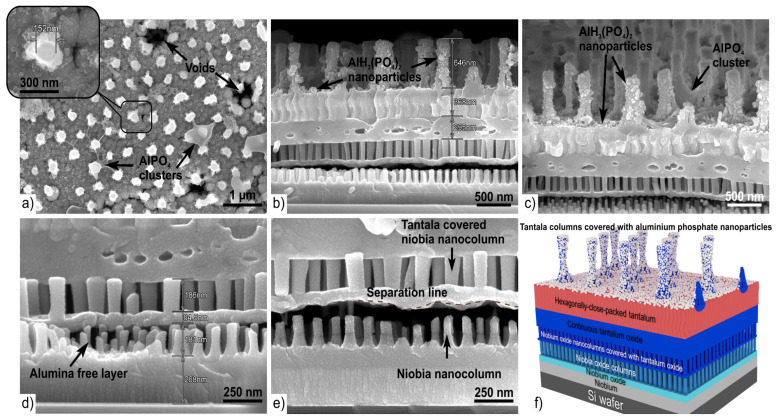
SEM surface (**a**), cross-fractured (**b**–**e**) and 3D schematic views (**f**) of the anodic two-level column-like 3D nanofilm with hexagonally-packed tantalum after 24 h long cold (23 °C) chemical etching in 50% aqueous orthophosphoric acid.

Figure 6a presents the second-level column-like 3D nanofilms of anodic tantala on the first-level niobia column-like 3D nanofilm. The structure of the second level differs from previously described column-like nanofilms of anodic tantala by the following features. Firstly, the base of each tantala column has a peculiar root structure consisting of 4–6 roots, as shown in Figure 6b. Secondly, the formation of column-like 3D nanofilms was carried out on nanostructured hexagonally-packed tantalum nanofilms. Thirdly, the continuous tantala layer appearing below the tantalum layer is characterized by high homogeneity and planarity without borders and transitions. The continuous tantala profile is clearly visible in Figure 6a. The presence of continuous tantala under the hexagonally-packed tantalum nanofilm can be explained by the following considerations. After the sputter-deposited tantalum film, voids were formed as described in Figure 7a.

At the stage of the second-level anodic formation, through the defects in the films, an electrolyte penetrated into the voids. The oxidation of the tantalum film in the voids occurred during the reanodizing step. The thickness of the formed continuous tantala corresponds to the 450 V reanodizing voltage at a volume growth factor of 1.8, which is confirmed by Ref. [45]. It should be noted that borders and transitions between grains appear on the surface of the tantalum layer even after reanodizing, as seen in Figure 6c. The presence of hexagonally-packed tantalum after reanodizing is explained by the fact that PAA cells are larger than tantalum grains.

### 3.4. Second-Level Morphology after Long Room Temperature Chemical Etching

In order to study in detail the structure of the two-level system with an array of niobia columns in the lower part and tantala columns in the upper part, it was necessary to remove the PAA in each layer of this system. The upper PAA, in which the tantala columns array grew, was dissolved quite quickly, while the lower PAA practically did not dissolve. To remove the lower PAA with niobia columns, we used long etching (24 h) of the PAA in 50% aqueous orthophosphoric acid at room temperature (~23 °C). Figure 7 shows the two-level column-like 3D nanofilms after this step. The differences between the hot short etching and the long cold one are clear when comparing Figure 6 and Figure 7. In the cross-fractured section of the two-level system subjected to the long cold etching (Figure 7b–e), one can see that the anodic PAA under the tantalum is completely dissolved. The continuous tantala film, lying on the first-level PAA of the first level, was detached from the lower part of the niobia columns, and the tops of the free-standing niobia columns are well distinguishable in the formed cracks.

In addition, as a result of the prolonged etching, a small rash of nanoparticles appeared on the tantala columns surface of the upper layer (Figure 7a–c). The etching reaction of alumina proceeds in several stages [46,47]. PAA on the surface quickly dissolves in the acid due to the large effective surface area, with the formation of Al^3+^ cations and oxygen-containing anions:

(2) $$Al_{2}O_{3}+6H^{+}=2Al^{3+}\left(aq\right)+3H_{2}O$$

The cations react with water molecules and form positively charged “aquasols” ${\mathrm{Al}}^{3+}\left(\mathrm{aq}\right)+{\;H}_{2}O\to \mathrm{Al}{\left({\mathrm{OH}}_{2}\right)}^{3+}\left(\mathrm{aq}\right)$. Next, the acid-base reaction of aquasols with aqueous phosphate anions occurs, and salts of aluminum hydrogen phosphates are formed:

(3) $$Al{\left(OH_{2}\right)}^{3+}+H_{2}PO_{4}{}^{-}+HPO_{4}{{}^{2}}^{-}\;\to AlH_{3}{\left(PO_{4}\right)}_{2}\cdot H_{2}O$$

Aluminum phosphates and hydrophosphates are condensed into nanoparticles and do not bind to each other and remain suspended, due to the small amount of dissolved alumina and a relatively large volume of solution. After keeping in solution for 24 h at room temperature, nanoparticles are adsorbed on the surface of the sample, as shown in Figure 7a–c, and are not washed off with thorough washing in distilled water. Similar processes occur during the formation of aluminum phosphate ceramics after prolonged storage in a solution of orthophosphoric acid [48].

### 3.5. Model of Film Growth during Anodizing and Reanodizing

Taking into consideration the previous experience in anodizing the Al/Ta couple [14,27], the EDX (Table 4) and SEM results (Figure 3, Figure 4, Figure 6 and Figure 7) show that we were able to fabricate incremental 3D oxide nanostructures at the nanostructured hexagonally-packed tantalum films, during their PAA-assisted anodizing followed by high-potential reanodizing.

**Figure 8:**
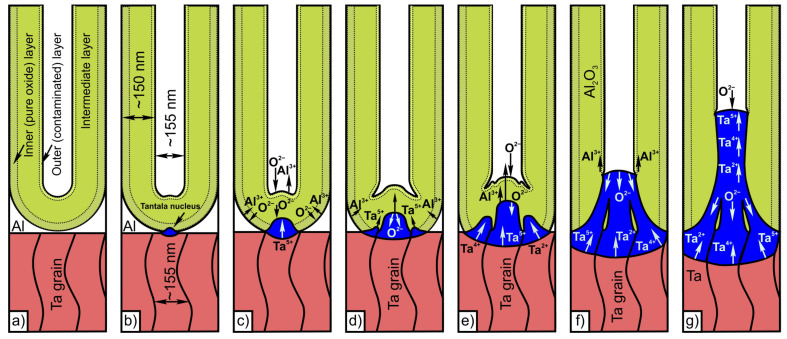
Ionic transport and anodic oxide growth during the PAA-assisted anodizing/reanodizing of the hexagonally-packed tantalum: (**a**) porous anodizing of the Al layer, (**b**) nucleation of anodic oxide from tantalum grain, (**c**) complete anodic oxidation of the grain surface, (**d**) nucleation of anodic oxide on tantalum neighboring grains, (**e**) growth of tantala from grains, (**f**) merging of tantala grains at pore bases, (**g**) beginning of the hexagonally-packed tantalum reanodizing, and steady-state growth of tantala during the reanodizing.

The main phases of the second-level growth are depicted in Figure 8, showing the development of anodic oxide nanostructures at the nanostructured hexagonally-packed tantalum films. According to the model, at the interface between the tantalum grain/alumina barrier layer, immediately under the bottoms of the cells, alumina touches the surface of the grain of nanostructured tantalum film. The O^2−^ ions migrating inwards through the alumina barrier layer are continuously injected into the grain of the tantala layer and, simultaneously, the barrier layer is continuously eliminated at the above interface, due to the dissociation of Al-O bonds under the high electric field (Figure 8b). The O^2−^ ions released from the dissociated barrier layer at the interface between the grain of hexagonally-packed tantalum/alumina barrier layer are also injected into the grain of tantala layer, while the released Al^3+^ ions migrate outwards through the remaining barrier layer and are mostly expelled in the electrolyte (Figure 8c). The O^2−^ ions injected into the tantala grain then migrate inwards and the tantalum grain is anodized normally to form a new oxide at the grain of the hexagonally-packed tantalum/tantala interface, while Ta^5+^ ions migrate outwards to form new oxide at the tantala grain/alumina barrier layer interface (Figure 8d).

Since the voltage is kept constant, the formation of new oxides at the tantala grain/alumina barrier layer interface and alumina/electrolyte interface is balanced exactly by elimination of the barrier layer, to maintain the oxide thickness necessary to sustain the voltage applied, accordingly to ionic resistance of the alumina-tantala combination. Oxidation of the hexagonally-packed tantalum extends to grain boundaries. Due to the high ionic resistance between the grains of hexagonally-packed tantalum, it is advantageous for ions to move through the alumina barrier layer to new grains. Thus, new growth points emerge around the oxidized grain (Figure 8e). The field distribution results in tantalum migrating towards the embryo-like column tips, rather than vertically across the film. The continued consumption of adjacent aluminum enhances expansion of the embryo-like columns horizontally (over the tantalum surface) and, simultaneously, is accompanied by further dissolution of the barrier layer until the ionic resistance of the embryos becomes comparable with that of the innermost parts of the alumina cells (pure PAA) remaining on the hexagonally-packed tantalum/alumina interface (Figure 8f). During reanodizing of the initially anodized Al/Ta bilayer (Figure 8g), the residual nanostructured tantalum is further oxidized and new column-like oxide grows continuously and is forced into the pores via cross-migration of tantalum and oxygen ions. In this case, the competition of ionic flow along different paths of other resistance continues and a continuous layer and the base of the columns keeps increasing in diameter. The inclusion of new grains of nanostructured tantalum during anodizing provides the material required to increase the height of the columns. The columns height is directly proportional to the anodizing voltage. The development of upper lower layers is governed by the PBR value of tantalum/tantala, the transport number of tantalum species, and the ratio of ionic resistances of the tantala columns and the alumina cladding. Figure 8g presents the section of an alumina cell in the anodized Al/Ta bilayer, pointing out layers with different ionic conduction. A difference can be observed between the outer cell wall, intermediate zone, and stoichiometric PAA in the bottom-most alumina cell zone (Figure 8a). The higher ionic conduction of the outer cell wall is due to physical defects, electrolyte species, bound water and protons penetrated into the oxide vacancies [14]. In the Al/Ta bilayer, most Al–O bonds of the outer cell wall dissociate in the high field [14] because of the high density of structural defects and the lower layer resistivity.

### 3.6. Optical Simulation for Biosensor Applications

**Figure 9:**
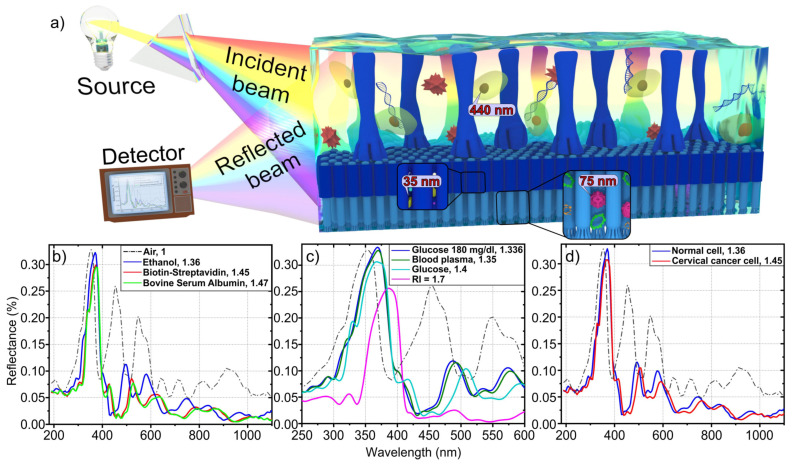
(**a**) 3D schematic view of the two-level column-like 3D anodic nanofilms biosensor. Reflectance responses of (**b**) biotin-streptavidin, bovin serum albumin components in blood, and ethanol; (**c**) different glucose concentrations in blood and blood plasma; (**d**) normal cell and cervical cancer cell. The legend shows the refractive index of the respective biomedical solutions.

As a suggested perspective for one possible key application of the two-level 3D anodic nanofilms demonstrated here, we next show that they can be promising for use in label-free optical biosensor devices, for example, aiming at the detection of viruses such as COVID-19. Label-free biosensors have certain advantages over marker biosensors, such as speed, economy, reliability and high sensitivity. Label-free optical biosensors are usually based on changes in the RI, depending on the concentration and type of biological target. When a target hits the biosensor, the RI of the surrounding medium changes, which leads to a shift in the resonant frequency of a patterned sensor surface. Operation of an optical biosensor based on the two-level column-like 3D anodic nanofilms can also be based on the registration of a shift in reflectance peaks, similar to previously PAA-based ones, yet with much higher versatility in optical properties tuning, thanks to the complex nanostructure and combination of different oxides. The 3D schematic view of two-level 3D anodic nanofilms biosensors is presented in Figure 9a. Due to the multi-level and dimensionality of the system, a wide range of optical properties can be obtained, in addition to the intrinsic material’s inertness to chemicals, potentially providing increased selectivity and sensitivity to a wide range of concentrations and types of biological targets.

In our system, the different biological target solutions filling the voids will differently change the RI of the structure as compared to when they are filled by air only. The two-level column-like 3D anodic nanofilms biosensor consists of three types of nanocolumn arrays: tantala columns with 90–145 nm diameter, as average value of *D_low_* and *D_hign_* from Table 3, niobia columns coated by tantala with 95 nm diameter, and niobia columns with 57 nm diameter. Each array provides a different reflectance peak (at wavelengths of 335, 456, and 548 nm, respectively), and at the same times makes it possible to immobilize a specific type of biological target, allowing for selectivity. Specifically, the tantala arrays may immobilize biological objects with dimensions of ~440 nm and high density biomedical liquid; the niobia-covered tantala arrays may immobilize biological objects with dimensions of ~35 nm and low density biomedical liquid; the niobia arrays may immobilize biological objects with dimensions of ~75 nm and medium density biomedical liquid. Overall, the proposed biosensing system can provide a high level filling factor.

Figure 9b–d show the results of the optical sensitivity simulation of two-level 3D anodic nanofilms to biomedical objects and liquids. Morphological and material parameters for the simulation were taken from Table 1, Table 2 and Table 3 and Refs [49,50,51]. Only column-like layers were simulated. The results showed a strong response in the form of reflection peaks shift of the two-level 3D anodic nanofilms to biotin-streptavidin and bovin serum albumin components in blood and ethanol (Figure 9b), cervical cancer cells (Figure 9d), and glucose in the blood (Figure 9c).

Additional optical performance simulation results of two-level 3D anodic nanofilms are presented in the Supplementary Materials in Figures S1–S6.

## 4. Conclusions

The 3D two-level column-like nanofilms have been synthesized via multi-step electrochemical anodizing of sputter-deposited Al/Nb and Al/Ta metal layers. The film growth, achieved so far in oxalic, tartaric and boric acid aqueous solutions at room temperature, proceeds in consecutive steps involving the formation of a PAA layer on niobium of the first-level, local oxidation of the niobium underlayer through the alumina barrier layer, lengthening of the oxide within the PAA with the simultaneous growth of a relatively uniform niobia layer, the partial removal of PAA by chemical etching, the sputter-deposition of Al/Ta layers on the first-level top, the formation of a PAA layer on tantalum of the second-level, and the lengthening of the oxide within the PAA pores with the simultaneous growth of a relatively uniform tantala layer. As a result of the work, the following points have emerged:

1. A first-level column-like 3D nanofilm, in which an array of niobia nanocolumns (∼57 nm diameter and ∼130 nm distance) penetrate the pores of a ∼163-nm-thick anodic alumina film, emerging above the film at ∼281 nm, has been fabricated by anodizing Al/Nb layers coated on silicon. The columns have the same structure and thickness along the entire length, in contrast to the skittle-like columns formed at higher voltages.
2. The first-level column-like 3D nanofilm was coated with an Al/Ta bilayer. The tantalum layer covered the remaining aluminum layer and the walls of the niobia columns, and was nanostructured into a single continuous layer on the surface of these columns, repeating their arrangement. For the first time, hexagonally-packed tantalum was obtained, repeating the hexagonal structure of PAA on the surface of niobia columns.
3. A second-level column-like 3D nanofilm was formed by anodizing the hexagonally-packed tantalum nanofilm through the PAA. The base of each column of tantala has a characteristic structure consisting of 4–6 roots. The first appearance below the layer of the continuous tantala layer is characterized by high homogeneity and planarity without borders and transitions. The experimental data justified the proposed model of a second-level columnar 3D nanofilm grown from hexagonally-packed tantalum nanofilm, involving field-assisted ionic transport and proper solid-state reactions.
4. Long-term chemical etching of the two-level column-like 3D nanofilms for ~24 h in 50% aqueous phosphoric acid resulted in a complete removal on the first- and the second-level of PAA, leading to the formation and condensation of aluminum phosphates and hydrophosphates into nanoparticles, with their subsequent precipitation.
5. The fabrication process reported here can result into two-level column-like 3D nanofilms with embedded oxide nanocolumns of various length, width, type of oxides and their relative population, all of which can be tuned by the respective PAA previously formed, according to the respective conditions. These two-level column-like 3D nanofilms appear promising for applications in photonic crystals for full-color displays, chemical sensors and biosensors, solar cells and thermoresponsive shape memory polymer devices. This fabrication technique makes it possible to integrate these dielectric layers with anodically fabricated planar thermoresistors, capacitors and nanowires, as demonstrated in former reports.

## Figures and Tables

**Table 2:** Morphological parameters of the first-level nanostructures.

Parameter	Value
Aluminum thickness (nm)	1200
Niobium thickness (nm)	300
Anodizing/reanodizing voltage (V)	53/450
PAA thickness (nm)	163
Average diameter of PAA cell (nm)	130
Niobium metal thickness (nm)	198
Continuous niobia thickness (*H_low_*, nm)	122
Height of niobia columns (*H_up_*, nm)	444
Distance between column centers (nm)	129
Average diameter of niobia columns (nm)	57

**Table 3:** Morphological parameters of second-level nanostructures.

Parameter	Value
Continuous tantalum layer height (nm)	426
Average tantalum grain diameter (nm)	120
Distance between continuous tantalum and Ta layer on PAA (nm)	340
Anodizing/reanodizing voltage (*V*)	200/450
Height of tantala columns (*H_up_*_,_ nm)	635
Minimum diameter of column (*D_low_*, nm)	90
Maximum diameter of columns (*D_high_*, nm)	145
Diameter of tantala columns base (nm)	359
Distance between column centers (nm)	556
Continuous structured Ta metal layer (nm)	364
Continuous tantala layer (nm)	282
Tantala layer on PAA (nm)	87

**Table 4:** EDX point analysis of the hexagonally-packed tantalum.

Element	Atomic Number	Series	Unn.C [wt.%]	Norm.C [wt.%]	Unn.C [at.%]	Error [wt.%]
**Carbon**	6	K-series	10.76	11.40	57.57	1.7
**Oxygen**	8	K-series	3.03	3.21	12.16	0.6
**Aluminum**	13	K-series	0.81	0.86	1.92	0.1
**Tantalum**	73	L-series	79.79	84.54	28.34	2.7
		Total:	94.39	100.00	100.00	

## Data Availability

The information that supports the findings of this investigation is available from A.P., upon reasonable request.

## Supplementary Materials

The following supporting information can be downloaded at: https://www.mdpi.com/article/10.3390/ma16030993/s1, Figure S1 is 3D animation representation of the two-level column-like 3D anodic nanofilms bio-sensor operation principles, Figure S2 is FDTD simulation of two-level 3D column-like nanofilms ZY view by Lumerical Inc. software ver. 8.24.2502. Figure S3 is FDTD simulated transmittance and reflectance spectra as a function of wavelength for the two-level 3D column-like nanofilms with marked three points of maximums on the reflection curve at 355 (S4), 456 (S5), 548 nm (S6) wavelength and Poynting vector, respectively, Figures S4–S6.
