# Supplementary material for: Two-Level 3D Column-like Nanofilms with Hexagonally–Packed Tantalum Fabricated via Anodizing of Al/Nb and Al/Ta Layers—A Potential Nano-Optical Biosensor
Source: Materials (Basel). 2023 Jan 21;16(3):993. doi: 10.3390/ma16030993 (PMC9918218; doi:10.3390/ma16030993)
Supplement: Supplementary file 1 [file materials-16-00993-s001.zip › materials-2137130-supplementary.pdf]

materials

Supplementary Materials: Two-level 3-D Column-like Nanofilms with Hexagonally-Packed Tantalum Fabricated via Anodizing of Al/Nb and Al/Ta Layers – A Potential Nano-optical Biosensor

Andrei Pligovka [1,2,*], Andrei Lazavenka [1,2], Ulyana Turavets [1,2], Alexander Hoha [1,2], and Marco Salerno [3]

1 Research and Development Laboratory 4.1 "Nanotechnologies", Belarusian State University of Informatics and Radioelectronics, 6 Brovki Str., 220013 Minsk, Belarus
2 Department of Micro- and Nanoelectronics, Belarusian State University of Informatics and Radioelectronics, 6 Brovki Str., 220013 Minsk, Belarus
3 Institute for Globally Distributed Open Research and Education (IGDORE) and Institute for Materials Science, Max Bergmann Center of Biomaterials, Technische Universität Dresden, Budapester Str. 27, 010169 Dresden, Germany

* Correspondence: pligovka@bsuir.by; Tel.: +375-44-730-95-81; Fax: +375-17-293-23-56

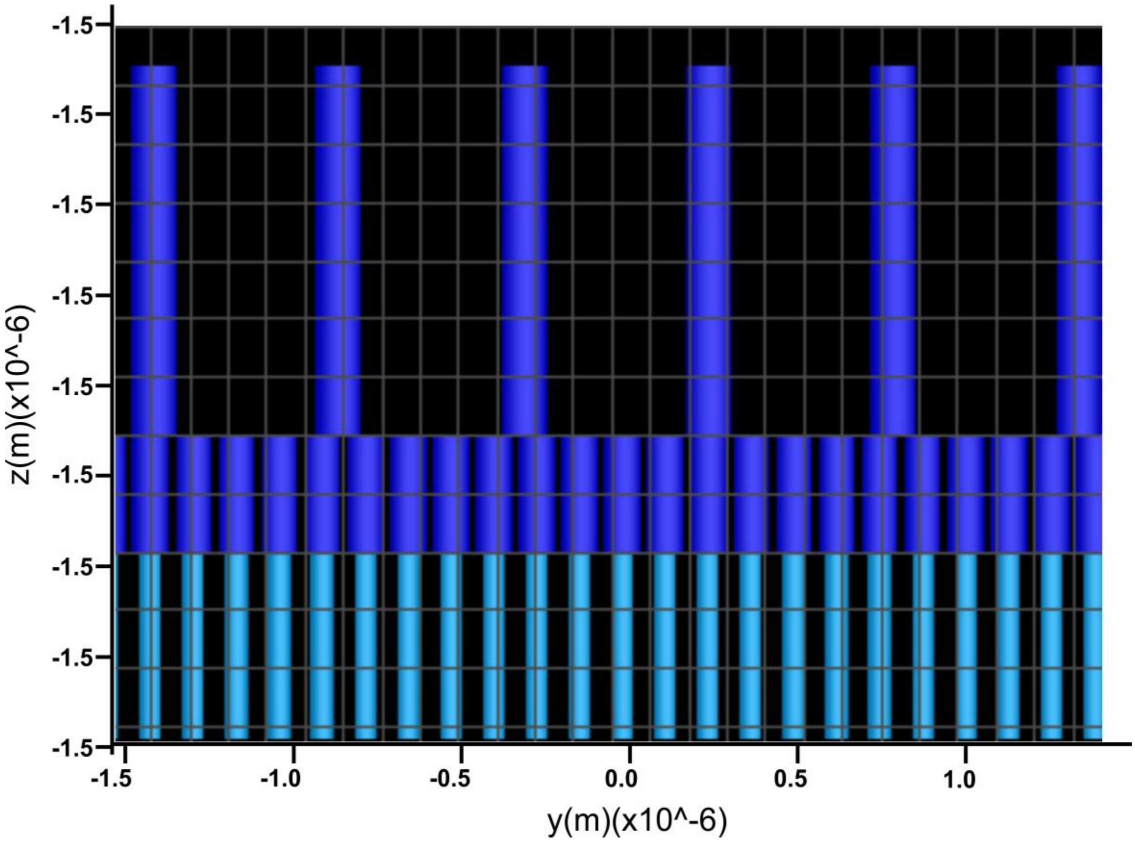

Figure S1. FDTD simulation of two-level 3-D column-like nanofilms ZY view by Lumerical Inc. software.

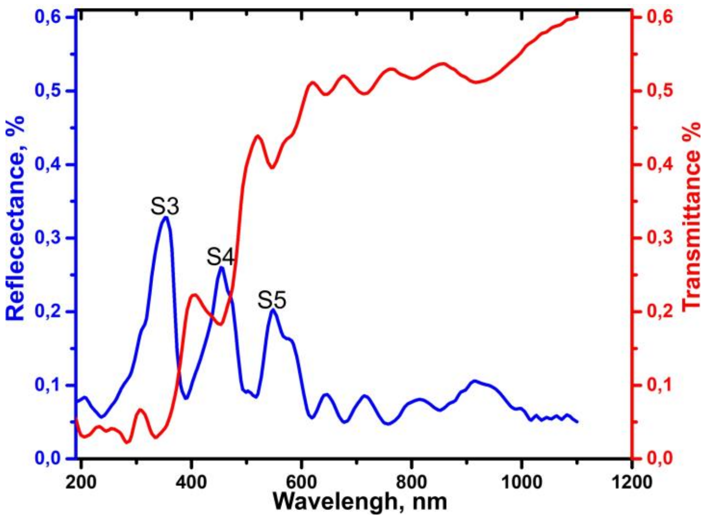

Figure S2. FDTD simulated transmittance and reflectance spectra as a function of wavelength for the two-level 3-D column-like nanofilms with marked three points of maximums on the reflection curve at 355 nm(S3), 456 nm(S4), 548 nm(S5) wavelength.

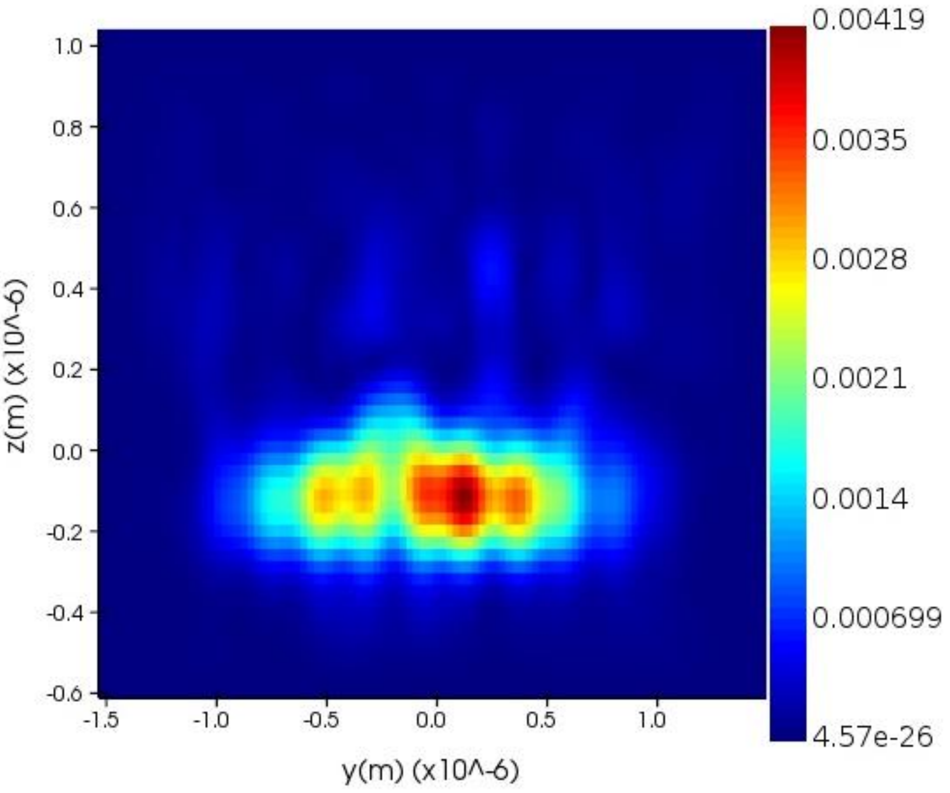

Figure S3. Poynting vector as the electromagnetic field distribution (control by YZ-plane reflectance monitor) for the first maximum of reflectance on 355 nm (S3) wavelength according to the reflectance curve on Figure S2.

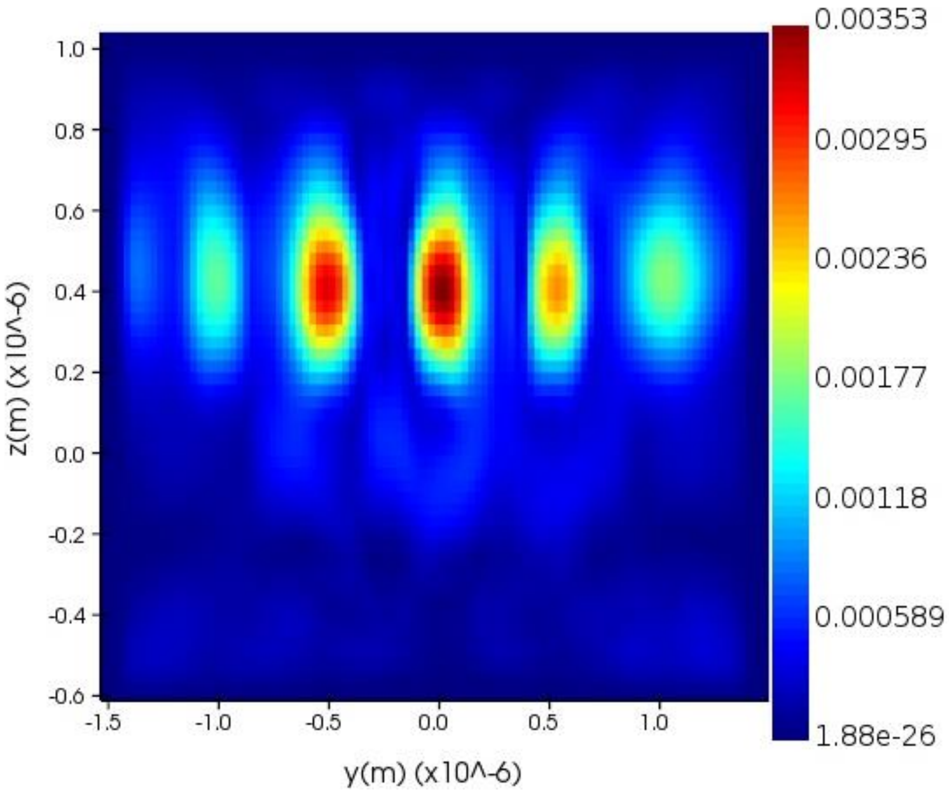

Figure S4. Poynting vector as the electromagnetic field distribution (control by YZ-plane reflectance monitor) for the second maximum of reflectance on 456 nm(S4) wavelength according to the reflectance curve on Figure S2.

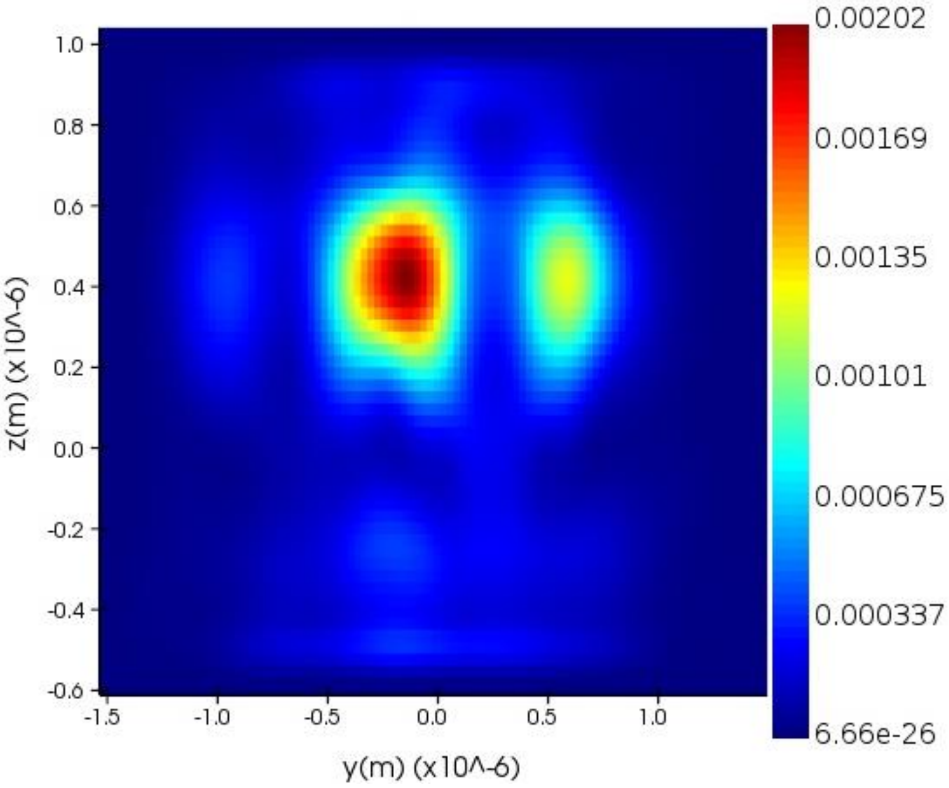

Figure S5. Poynting vector as the electromagnetic field distribution (control by YZ-plane reflectance monitor) for the third maximum of reflectance on 548 nm(S5) wavelength according to the reflectance curve on Figure S2.
